# Supplementary material for: Best practices for spatial language data harmonization, sharing and map creation—A case study of Uralic
Source: PLoS One. 2022 Jun 8;17(6):e0269648. doi: 10.1371/journal.pone.0269648 (PMC9176854; doi:10.1371/journal.pone.0269648)

North Khanty

The assignment is to define as precisely as possible:

1) the maximal distribution of the speaker areas (=classic speaker area, approximately at the beginning of 20th century)
2) the geographical location of the current speaker areas

Sources

Abondolo, D. (toim.) 1998. The Uralic Languages. London, Routledge. 619 s. (Map iv)
Grünthal, R. & Salminen, T. (edited) 1993. Geographical distribution of the Uralic Languages. Finno-Ugrian Society.
Haarmann, H. 1974: 215 (Map iii In Abondolo, D. (ed.) 1998. The Uralic Languages. London, Routledge.)
Lytkin, V. I., Maytinskaya, K. E. & Rédei, K. (edited) 1976. The Fundamentals of Fenno-Ugric Linguistics. The Academy of Sciences of the USSR 3. Moscow, Nauka.

Maps are based on these sources. It is necessary to know:

a) how well these are matching up to current opinion of the experts
b) which source is the most accurate or correct
c) and if it is needed how borders should be modificated

Possible modifications can be done for example by drawing new borders in printed version or straight to the file. One way is to give email comments (and/or tell the source which should be used to). There are many draft maps included but you can make your changes to one or more map, just choose the most convenient way on you case. If changes are not needed I hope you can also mention that.

Comments and detailed questions

I have understood that rivers (and their drainage basins) are important when defining the distribution of Khanty speakers. I am sorry that maps are not a fully comprehensive in this regard and hopefully this not complicating the interpretation.

When was the most extensive (maximal) distribution of North Khanty speaker area?

How would you define current speaker area of North Khanty? If it is difficult to do you can just name important villages and other settlements.
Does North Khanty have new important speaker areas outside traditional region? You can mention for example towns and other settlements outside the traditional area.

What is a native term for North Khanty?

What is your opinion, should South Khanty also be included in the map of Oxford book? If you answered yes, should it be presented rather with North or East Khanty?

What kind of other information final map would be incorporated? Essential place names? More detailed visualisations of different dialects?

Other comments?

Whole speaker area of Khanty

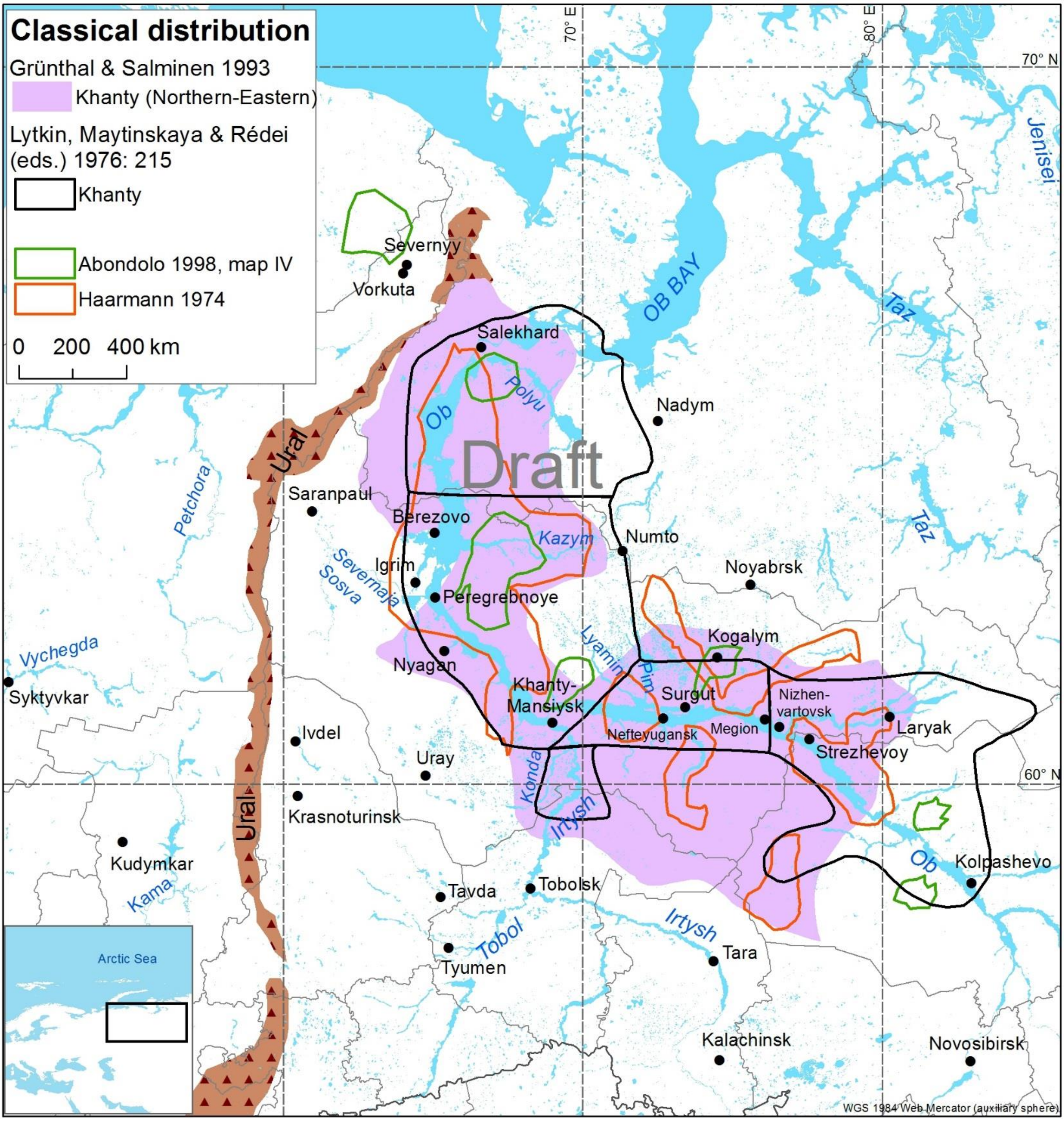

Whole area - different Khanty dialect groups/dialects

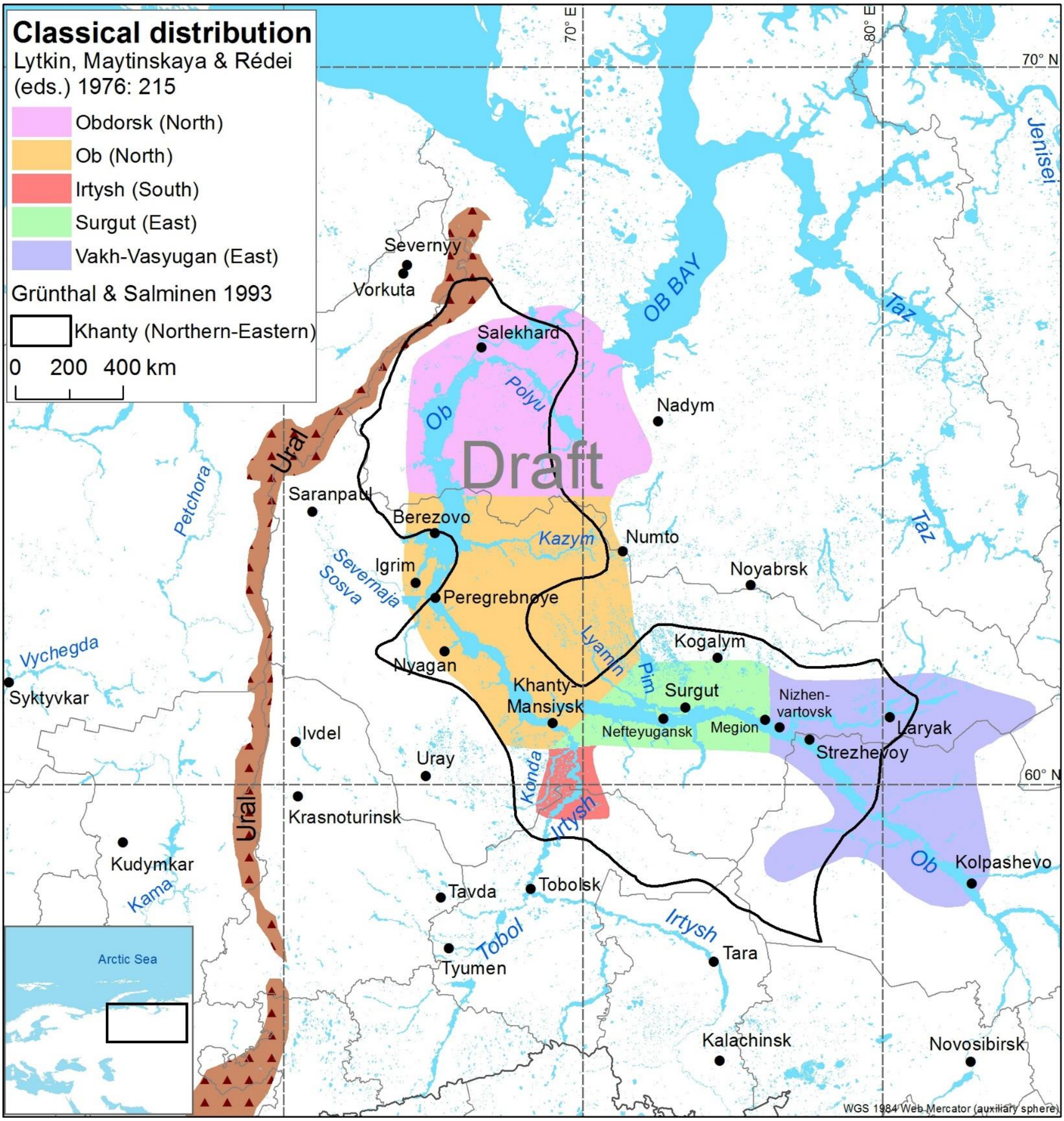

Whole area, current distribution?

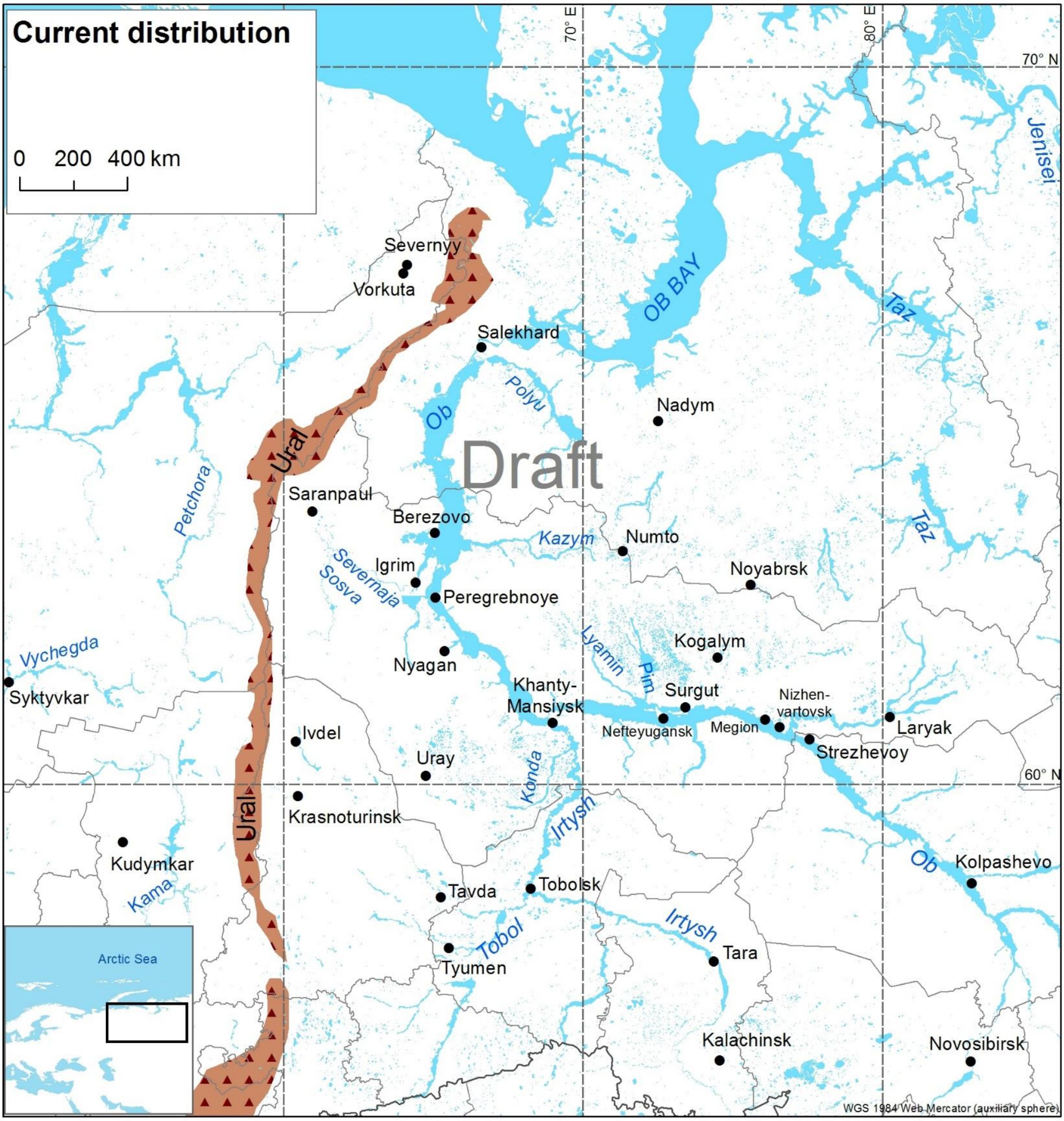

Northern part more closely

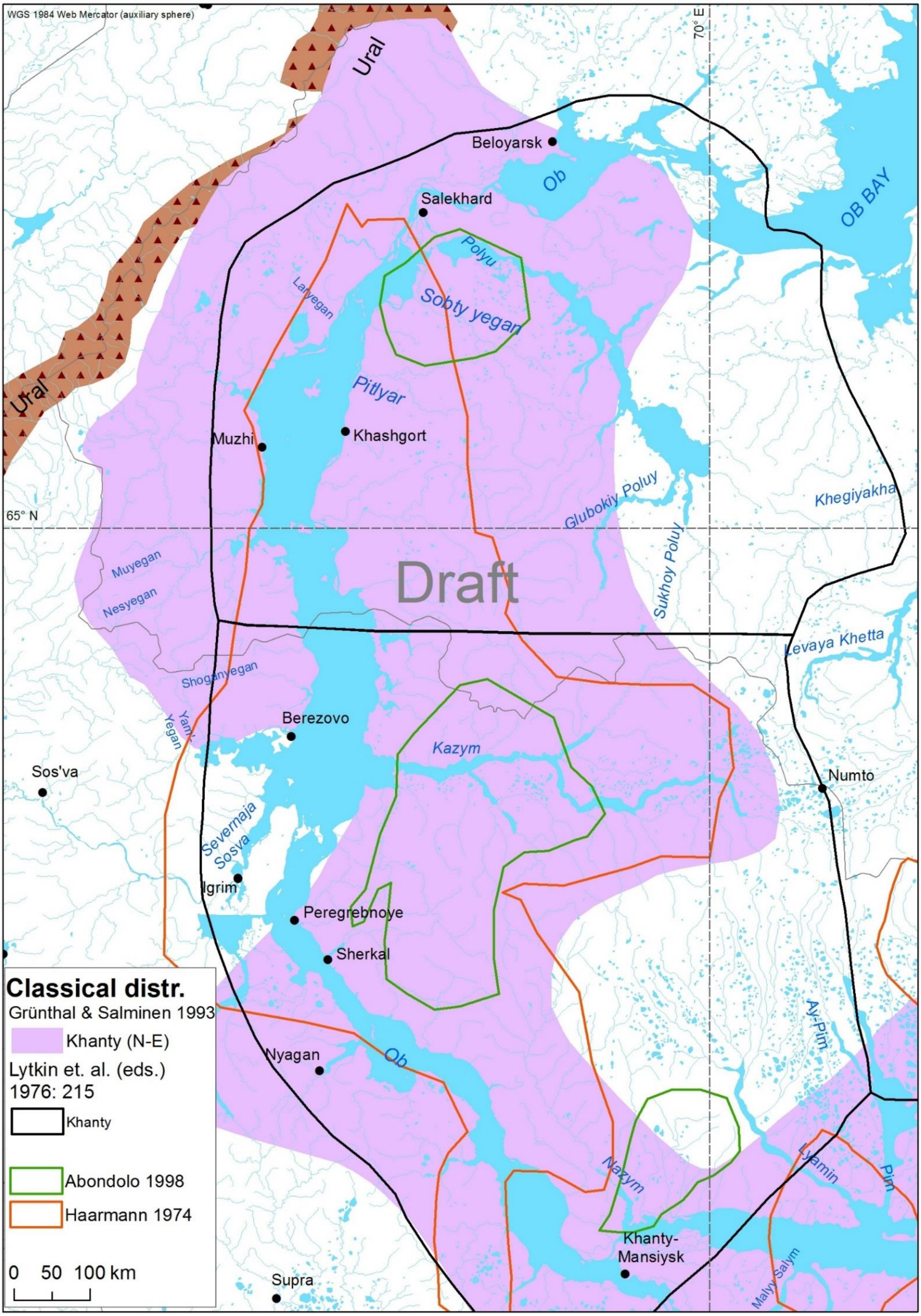

Northern part more closely

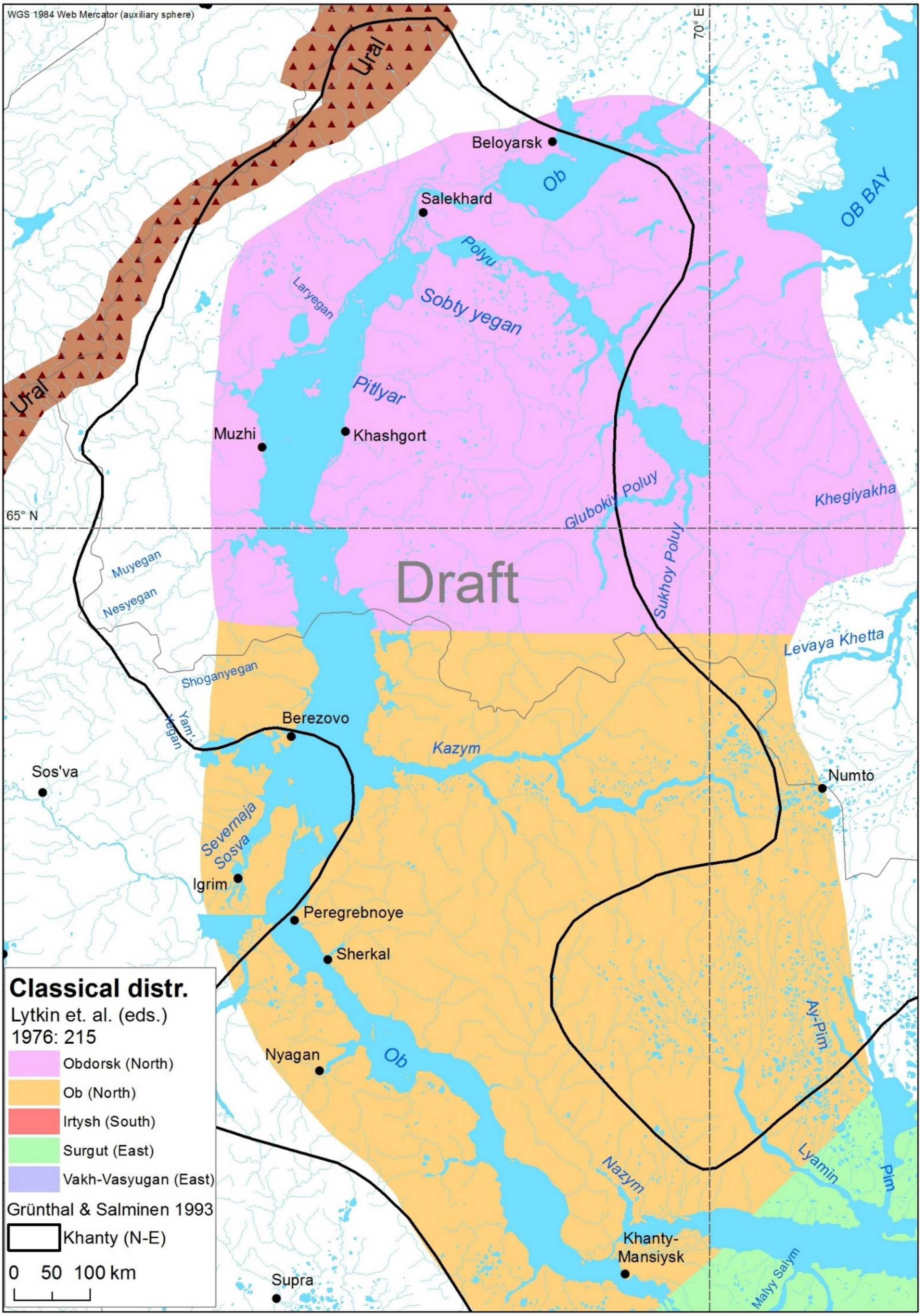

Northern part, current distribution?

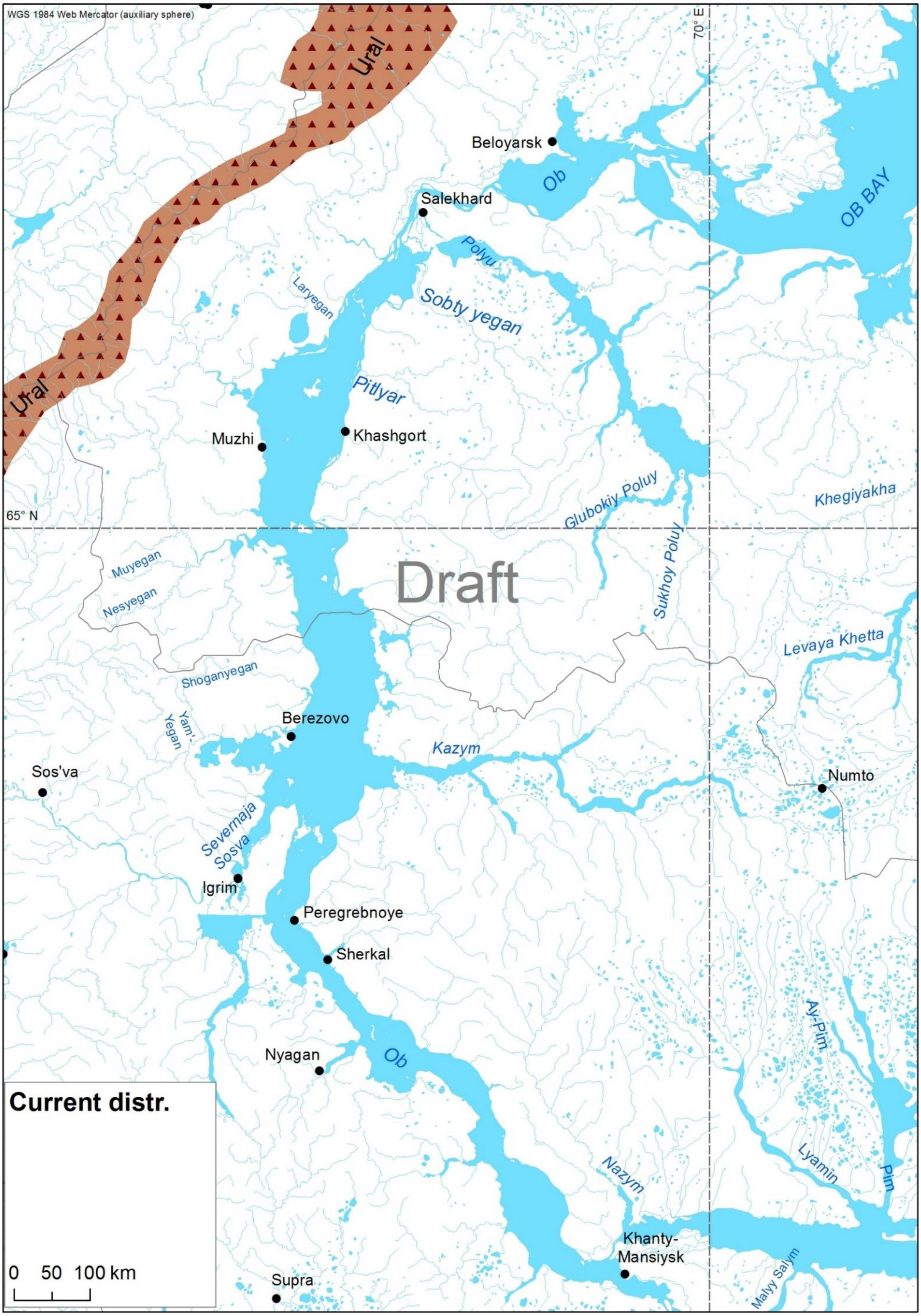

Southern part more closely

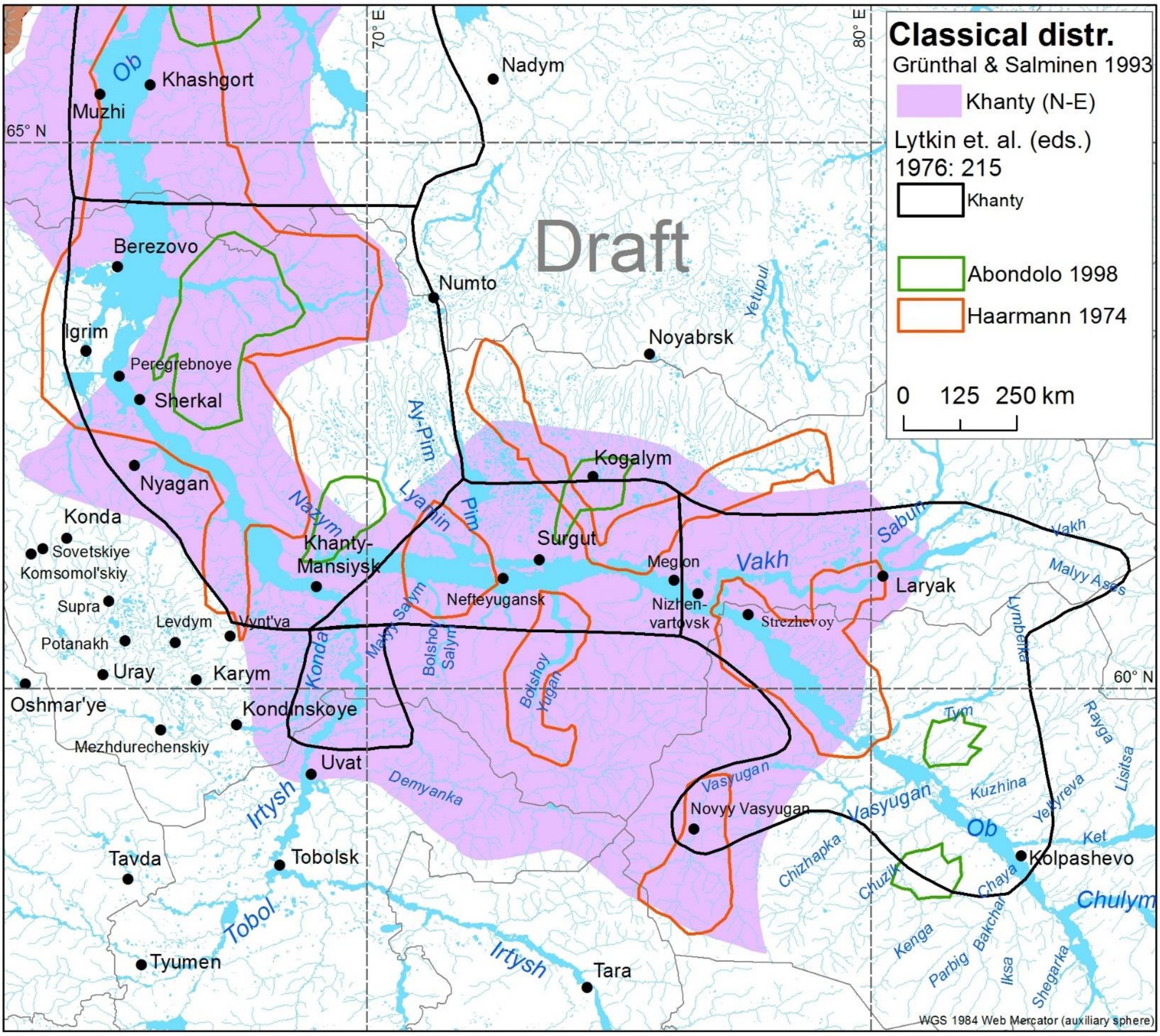

Southern part more closely

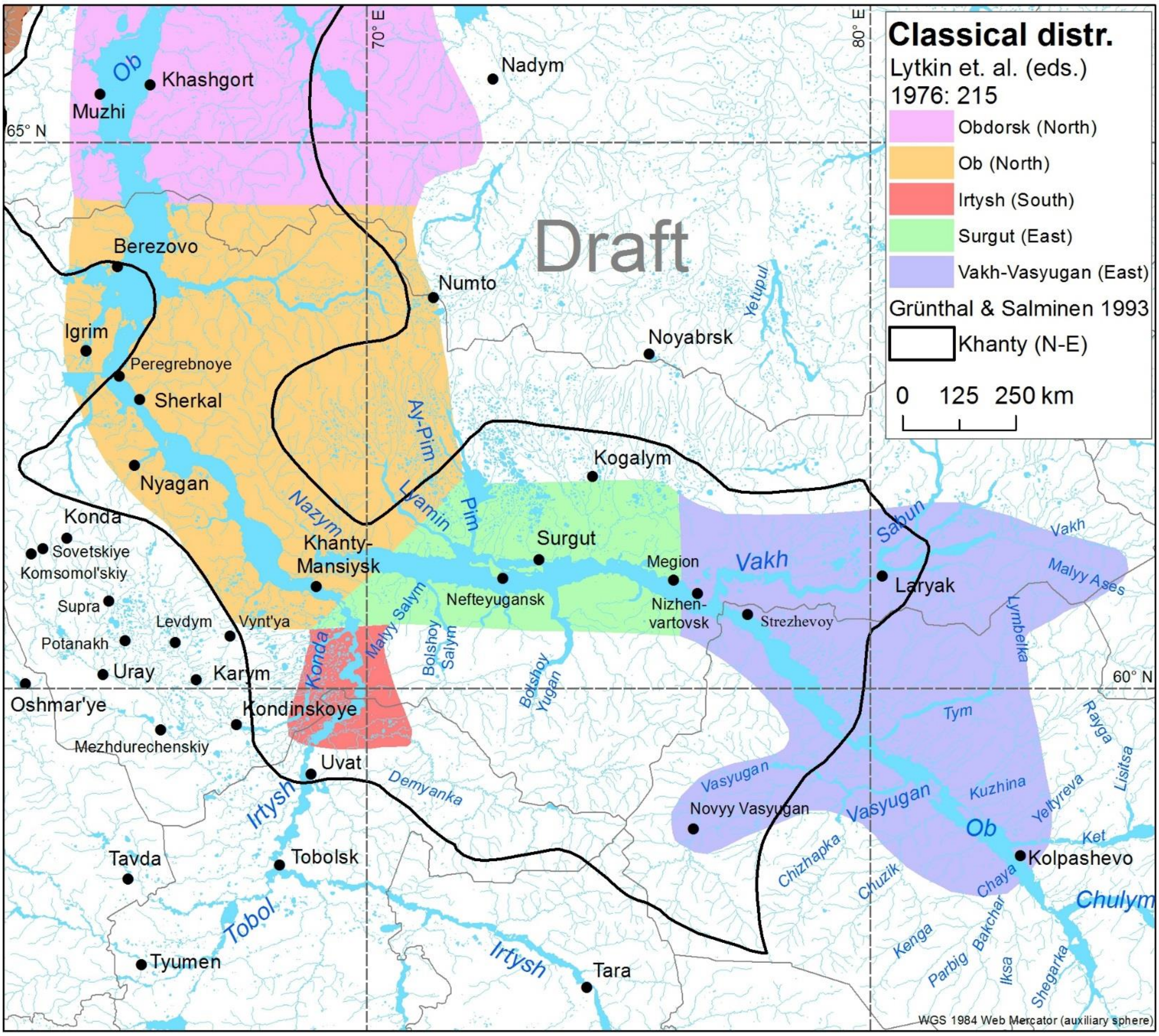

Southern part, current distribution?

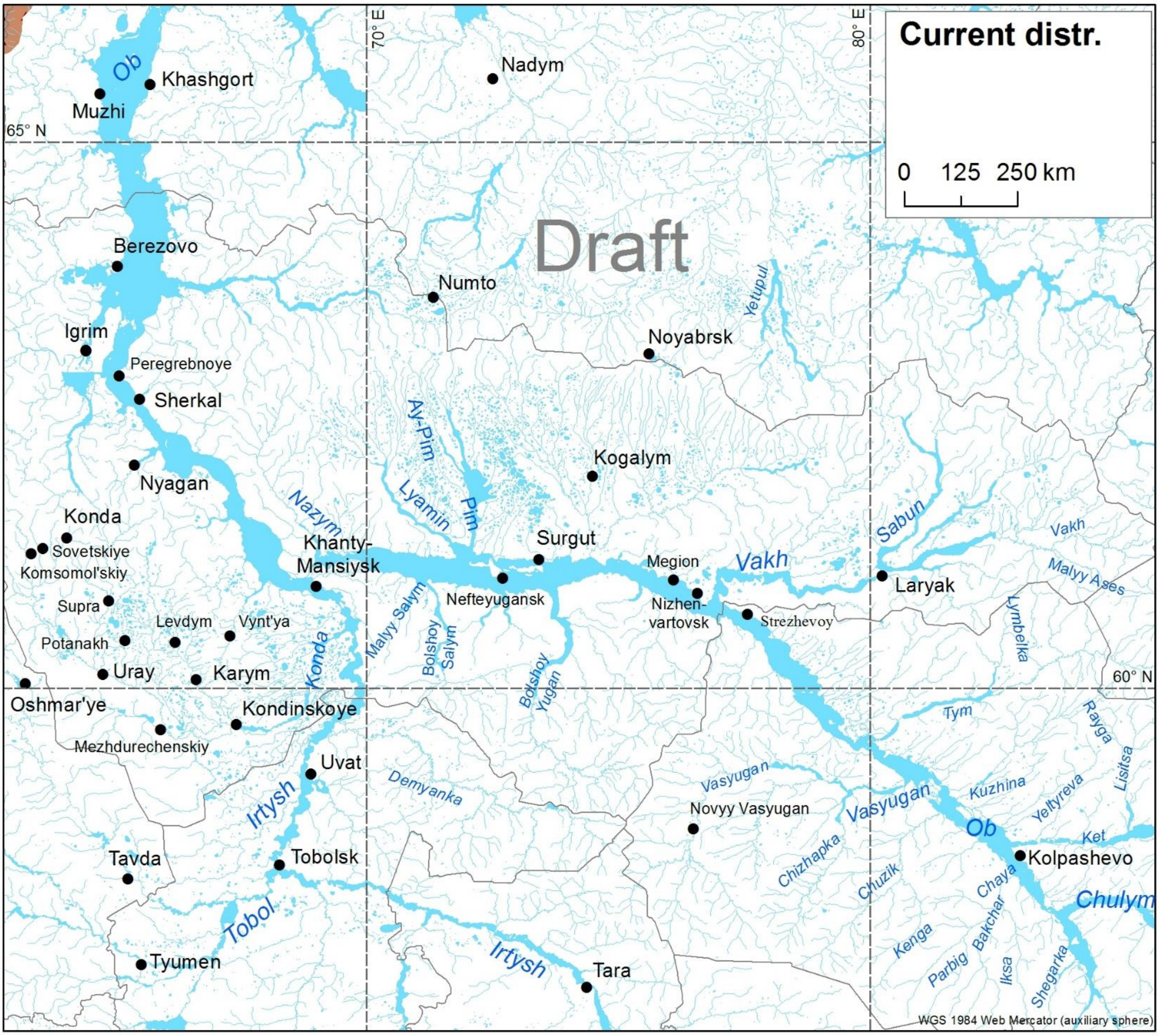

Supplement: S1 Appendix — Similar query was sent to each responsible author(s) of the particular language chapters of The Oxford Guide to the Uralic Languages. These expert evaluations were created to collect up-to-date information of past and present geographical distribution of Uralic languages. (PDF) [file pone.0269648.s001.pdf]
