# Supplementary material for: Best practices for spatial language data harmonization, sharing and map creation—A case study of Uralic
Source: PLoS One. 2022 Jun 8;17(6):e0269648. doi: 10.1371/journal.pone.0269648 (PMC9176854; doi:10.1371/journal.pone.0269648)
Supplement: S2 Appendix — Rantanen T, Vesakoski O, Ylikoski J, Tolvanen H. Geographical database of the Uralic languages. Version v1.0.; 2021. Database: Zenodo [Internet]. Available from: http://doi.org/10.5281/zenodo.4784188. (DOCX) [file pone.0269648.s002.docx]

**S1 Appendix.** Rantanen T, Vesakoski O, Ylikoski J, Tolvanen H. Geographical database of the Uralic languages. Version v1.0.; 2021. Database: Zenodo [Internet]. Available from: <http://doi.org/10.5281/zenodo.4784188>
