# Supplementary material for: Best practices for spatial language data harmonization, sharing and map creation—A case study of Uralic
Source: PLoS One. 2022 Jun 8;17(6):e0269648. doi: 10.1371/journal.pone.0269648 (PMC9176854; doi:10.1371/journal.pone.0269648)
Supplement: S1 Table — Language distributions are based on the published studies (original) and separate expert evaluations (expert) done in collaboration with the authors of The Oxford Guide to the Uralic Languages. Some original studies do not separate subgroups of language branches, which is the reason to use branch or general names in the ‘Language’ column (labelled in italics) in some cases, for example ‘Mordvin’ or ‘Khanty’. Branches: Saami (I), Finnic (II), Mordvin (III), Mari (IV), Permic (V), Mansi (VI), Khanty (VII), Hungarian (VIII), Samoyedic (IX). *Skolt Saami: Distribution after resettlement in 1950s. **Livonian: Medieval and 1900s distributions. (DOCX) [file pone.0269648.s003.docx]

**S1 Table. Number of distributions per language and time period in the geospatial datasets.**

|  |  | Traditional | | | Current | | | **All** |
| --- | --- | --- | --- | --- | --- | --- | --- | --- |
| **Branch** | **Language** | original | expert | sum | original | expert | sum |  |
| I | South Saami | 4 | 1 | 5 | 0 | 0 | 0 | **5** |
| I | Ume Saami | 4 | 1 | 5 | 0 | 0 | 0 | **5** |
| I | Pite Saami | 4 | 1 | 5 | 0 | 0 | 0 | **5** |
| I | Lule Saami | 3 | 1 | 4 | 0 | 0 | 0 | **4** |
| I | North Saami | 4 | 1 | 5 | 0 | 0 | 0 | **5** |
| I | Aanaar Saami | 4 | 1 | 5 | 0 | 0 | 0 | **5** |
| I | Skolt Saami | 7 | 1 | 8 | 0 | 1* | 1 | **9** |
| I | Kildin Saami | 5 | 1 | 6 | 0 | 0 | 0 | **6** |
| I | Akkala Saami | 4 | 0 | 4 | 0 | 0 | 0 | **4** |
| I | Ter Saami | 5 | 0 | 5 | 0 | 0 | 0 | **5** |
| II | Finnish | 3 | 1 | 4 | 0 | 0 | 0 | **4** |
| II | Karelian | 2 | 1 | 3 | 1 | 1 | 2 | **5** |
| II | Ludic | 2 | 1 | 3 | 0 | 1 | 1 | **4** |
| II | Veps | 2 | 1 | 3 | 0 | 1 | 1 | **4** |
| II | Ingrian | 2 | 1 | 3 | 0 | 1 | 1 | **4** |
| II | Votic | 2 | 1 | 3 | 0 | 1 | 1 | **4** |
| II | North Estonian | 2 | 1 | 3 | 0 | 0 | 0 | **3** |
| II | South Estonian | 1 | 1 | 2 | 0 | 0 | 0 | **2** |
| II | Livonian** | 1 | 2 | 3 | 0 | 0 | 0 | **3** |
| III | Erzya | 3 | 1 | 4 | 0 | 0 | 0 | **4** |
| III | Moksha | 3 | 1 | 4 | 0 | 0 | 0 | **4** |
| *III* | *Mordvin* | 1 | 0 | 1 | 0 | 0 | 0 | **1** |
| IV | Hill Mari | 2 | 1 | 3 | 0 | 0 | 0 | **3** |
| IV | North-Western Mari | 1 | 1 | 2 | 0 | 0 | 0 | **2** |
| IV | Meadow Mari | 2 | 1 | 3 | 0 | 0 | 0 | **3** |
| IV | Eastern Mari | 1 | 1 | 2 | 0 | 0 | 0 | **2** |
| *IV* | *Mari* | 1 | 0 | 1 | 0 | 0 | 0 | **1** |
| V | Komi-Zyrian | 5 | 1 | 6 | 0 | 0 | 0 | **6** |
| V | Komi-Permyak | 5 | 1 | 6 | 0 | 0 | 0 | **6** |
| V | Yazva Komi | 3 | 1 | 4 | 0 | 0 | 0 | **4** |
| *V* | *Komi* | 0 | 1 | 1 | 0 | 0 | 0 | **1** |
| V | Udmurt | 3 | 0 | 3 | 1 | 1 | 2 | **5** |
| VI | North Mansi | 2 | 1 | 3 | 0 | 0 | 0 | **3** |
| VI | East Mansi | 2 | 1 | 3 | 0 | 0 | 0 | **3** |
| VI | West and South Mansi | 2 | 1 | 3 | 0 | 0 | 0 | **3** |
| *VI* | *Mansi* | 4 | 0 | 4 | 0 | 1 | 1 | **5** |
| VII | North Khanty | 0 | 1 | 1 | 0 | 1 | 1 | **2** |
| VII | East Khanty | 0 | 1 | 1 | 1 | 1 | 2 | **3** |
| VII | South Khanty | 0 | 1 | 1 | 0 | 0 | 0 | **1** |
| *VII* | *Khanty* | 5 | 0 | 5 | 0 | 0 | 0 | **5** |
| VIII | Hungarian | 1 | 0 | 1 | 0 | 1 | 1 | **2** |
| IX | Forest Nenets | 1 | 2 | 3 | 0 | 2 | 2 | **5** |
| IX | Tundra Nenets | 1 | 2 | 3 | 0 | 2 | 2 | **5** |
| IX | Nenets | 1 | 0 | 1 | 0 | 0 | 0 | **1** |
| IX | Forest Enets | 1 | 1 | 2 | 0 | 1 | 1 | **3** |
| IX | Tundra Enets | 3 | 1 | 4 | 0 | 1 | 1 | **5** |
| IX | Nganasan | 4 | 1 | 5 | 0 | 1 | 1 | **6** |
| IX | Northern Selkup | 1 | 1 | 2 | 0 | 1 | 1 | **3** |
| IX | Tomsk region Selkup | 0 | 1 | 1 | 0 | 1 | 1 | **2** |
| IX | Selkup | 2 | 0 | 2 | 0 | 0 | 0 | **2** |
| IX | Kamas | 3 | 1 | 4 | 0 | 0 | 0 | **4** |
| IX | Mator | 1 | 0 | 1 | 0 | 0 | 0 | **1** |
|  | **Sum** | **125** | **44** | **169** | **3** | **20** | **23** | **193** |
