# Supplementary material for: Best practices for spatial language data harmonization, sharing and map creation—A case study of Uralic
Source: PLoS One. 2022 Jun 8;17(6):e0269648. doi: 10.1371/journal.pone.0269648 (PMC9176854; doi:10.1371/journal.pone.0269648)
Supplement: S2 Table — Table shows how many dialects are presented per language as well as information of temporal coverage. Total number of language maps is 45. Main branches of Uralic languages are indicated as Roman numerals. *Meänkieli and Kven are seen here as separate languages, in S1 Table both instead belong to Finnish language. **Lule Saami is divided to three proper and three transitional dialects. ***Mordvin branch consists of five Erzya language dialects and four Moksha language dialects. (DOCX) [file pone.0269648.s004.docx]

**S2 Table.** **List of maps containing information on each Uralic language.**

| **Language or branch** | **No of dialects** | **Presented in a map** | **Traditional** | **Current** |
| --- | --- | --- | --- | --- |
| Saami (I) |  | 1; 0.1 | X |  |
| South Saami | 3 | 1.1; 1 | X |  |
| Ume Saami | 3 | 1.1; 1.2; 1 | X |  |
| Pite Saami |  | 1.2; 1.1; 1 | X |  |
| Lule Saami | 3+3** | 1.2; 1.3; 1 | X |  |
| North Saami | 4 | 1.3; 1.2; 1.4; 1.5; 1 | X |  |
| Aanaar Saami |  | 1.4; 1.3; 1.5; 1 | X |  |
| Skolt Saami | 2 | 1.5; 1.5; 1.3; 1.4; 1.6; 1 | X |  |
| Kildin Saami | 4 | 1.6; 1.5; 1 | X |  |
| Akkala Saami |  | 1.5; 1.6; 1 | X |  |
| Ter Saami |  | 1.6; 1 | X |  |
| Finnic (II) |  | 2; 0.1 | X |  |
| Finnish | 7 | 2.1; 2 | X |  |
| Meänkieli* |  | 2.1 | X |  |
| Kven* |  | 2.1 | X |  |
| Karelian | 4 | 2.2a; 2.2b; 2 | X | X |
| Ludic | 2 | 2.2a; 2.2b; 2 | X | X |
| Veps | 3 | 2.3; 2 | X | X |
| Ingrian | 4 | 2.4a; 2.4b; 2 | X | X |
| Votic | 3 | 2.4a; 2.4b; 2 | X | X |
| North Estonian | 5 | 2.5; 2.6; 2 | X |  |
| South Estonian | 4 | 2.6; 2.5; 2 | X |  |
| Livonian |  | 2.7; 2 | X |  |
| Mordvin (III) | 5+4*** | 3; 0.1 | X |  |
| Erzya | 5 | 3 | X |  |
| Moksha | 4 | 3 | X |  |
| Mari (IV) |  | 4; 0.1 | X |  |
| Hill Mari |  | 4 | X |  |
| North-Western Mari |  | 4 | X |  |
| Meadow Mari |  | 4 | X |  |
| Eastern Mari |  | 4 | X |  |
| Permic (V) |  | 5; 0.1 | X |  |
| Komi-Zyrian |  | 5.1a; 5.1b; 5 | X |  |
| Komi-Permyak |  | 5.1a; 5.1b; 5 | X |  |
| Yazva Komi |  | 5.1a; 5.1b; 5 | X |  |
| Udmurt | 4 | 5.2; 5 | X | X |
| Mansi (VI) |  | 6.1; 6.2; 6; 0.1 | X |  |
| North Mansi |  | 6.1; 6.2; 6 | X | X |
| East Mansi |  | 6.1; 6.2; 6 | X | X |
| West Mansi |  | 6.1; 6.2; 6 | X | X |
| South Mansi |  | 6.1; 6.2; 6 | X |  |
| Khanty (VII) |  | 6; 0.1 | X |  |
| North Khanty | 2 | 7.1a; 7.1b; 7.2a; 7.2b; 6 | X | X |
| East Khanty | 2 | 7.2a; 7.2b; 7.1a; 7.1b; 6 | X | X |
| South Khanty | 1 | 7.1a; 7.2a; 6 | X |  |
| Hungarian (VIII) | 10 | 8.1; 8.2a; 8.2b; 0.1 | X | X |
| Samoyedic (IX) |  | 9; 0.1 | X |  |
| Tundra Nenets |  | 9.1a; 9.1b; 9 | X | X |
| Forest Nenets |  | 9.1a; 9.1b; 9 | X | X |
| Tundra Enets |  | 9.2a; 9.2b; 9 | X | X |
| Forest Enets |  | 9.2a; 9.2b; 9 | X | X |
| Nganasan |  | 9.3; 9 | X | X |
| Northern Selkup | 6 | 9.4a; 9.4b; 9 | X | X |
| Tomsk region Selkup | 4 | 9.4a; 9.4b; 9 | X | X |
| Kamas | 2 | 9.5; 9 | X |  |
| Mator | 3 | 9.5; 9 | X |  |
